# Outcomes of total joint arthroplasty in patients with a history of native hip or knee septic arthritis: a systematic review and meta-analysis

**DOI:** 10.1186/s43019-026-00348-y

**Published:** 2026-08-31

**Authors:** Chang-Rack Lee, Dae-Hyun Park, Yong-Uk Kwon, Ji-Hun Park, Dong-Yun Lee

**Affiliations:** Department of Orthopaedic Surgery, Inje University Busan Paik Hospital, Busan, Republic of Korea

## Abstract

**Background:**

Whether a history of native hip or knee septic arthritis is associated with an increased risk of periprosthetic joint infection (PJI), aseptic revision, or reoperation after total joint arthroplasty (TJA) remains unclear. This meta-analysis aimed to evaluate whether patients with a history of native hip or knee septic arthritis (septic arthritis group) are at higher risk of PJI, aseptic revision, and reoperation after primary TJA than those without (nonseptic arthritis group). We hypothesized that a history of septic arthritis is associated with an increased risk of complications after TJA.

**Methods:**

A systematic literature search of MEDLINE, Embase, and the Cochrane Library was conducted to identify studies comparing the outcomes of primary TJA between patients with a history of native hip or knee septic arthritis and those without. Previous studies reporting postoperative infection, aseptic revision, or reoperation rates were included in the analysis. The septic arthritis group comprised patients with sequelae of childhood joint infection and those who underwent one- or two-stage TJA for active or previously treated septic arthritis. Random-effects meta-analyses were performed to calculate pooled risk ratios with 95% confidence intervals.

**Results:**

This research included seven studies, comprising 5842 arthroplasties in the septic arthritis group (total hip arthroplasty [THA]: 1351; total knee arthroplasty [TKA]: 4491) and 10,589 arthroplasties in the nonseptic arthritis group (THA: 5299; TKA: 5290). Patients with a history of septic arthritis were at significantly higher risk of postoperative PJI than those without, with substantial heterogeneity. The risk of aseptic revision did not significantly differ between the septic arthritis and nonseptic arthritis groups. However, patients with a history of septic arthritis were at significantly higher risk of reoperation.

**Conclusions:**

Patients with a history of native hip or knee septic arthritis are at significantly higher risk of postoperative infection and reoperation after total joint arthroplasty compared with those without a history of joint infection. Surgeons should be aware of the increased risk of complications in this high-risk population. Nevertheless, further studies should be performed to determine the optimal timing of arthroplasty after septic arthritis and to identify strategies that can reduce postoperative complications.

**Supplementary Information:**

The online version contains supplementary material available at https://doi.org/10.1186/s43019-026-00348-y.

## Background

Septic arthritis is an orthopedic emergency that requires prompt diagnosis and management, as delays in diagnosis or treatment can lead to extensive joint destruction [1]. The knee joint is the most commonly affected joint, accounting for approximately 50–60% of all septic arthritis cases. With an aging population and the growing number of patients with diabetes or immunocompromised conditions, the incidence of septic arthritis has risen [2, 3]. In addition, septic arthritis is considered a relatively common and important condition in pediatric orthopedics [4].

The fundamental principles of treating septic arthritis include comprehensive removal of infected tissues combined with appropriate antibiotic therapy. In knee or hip joint infection, various surgical treatment options including open synovectomy and arthroscopic management, have been proposed for the resection of infected tissues. Despite appropriate antibiotic therapy and surgical treatment, some patients with septic arthritis subsequently develop degenerative arthritis and require total joint arthroplasty (TJA). Further, in cases of knee or hip joint infection accompanied by advanced arthritis, two-stage TJA using an antibiotic-impregnated articulating or static cement spacer is occasionally performed [5–7].

Total knee arthroplasty (TKA) and total hip arthroplasty (THA) are effective treatment options for patients with end-stage arthritis of the knee or hip joint, with a long-term survivorship of > 90% [8]. However, several studies have reported that patients with a history of native knee or hip septic arthritis have a higher rate of complications, including periprosthetic joint infection (PJI), after TKA or THA than those without [9, 10]. Nevertheless, studies specifically addressing native hip or knee septic arthritis preceding TJA remain limited. To date, no systematic review or meta-analysis has quantitatively synthesized these comparative studies. Accordingly, this meta-analysis was undertaken to provide a quantitative synthesis of the currently available comparative evidence.

This meta-analysis aimed to evaluate whether patients with a history of native knee or hip septic arthritis are at higher risk of postoperative infection, aseptic revision, and reoperation after TJA compared with those without. We hypothesized that patients with a history of native knee or hip septic arthritis are at higher risk of complications after TJA.

## Methods

### Literature search and information sources

This study was conducted in accordance with the guidelines of the Preferred Reporting Items for Systematic Reviews and Meta-Analyses (PRISMA) statement and was based on the Cochrane review methods. A literature search was performed using the databases most commonly used in the medical field, including PubMed, EMBASE, and the Cochrane Library, from the date of inception to 7 November 2025. An information retrieval expert (medical librarian) conducted a structured search of research data based on our methodology. Online Appendix Table 1 presents the search protocol. After the initial database search, the reference lists of relevant articles were manually reviewed to identify additional eligible studies. Restrictions in terms of language or year of publication were not applied during the literature search. As this study conducted a systematic review of previously published literature, approval from an institutional review board and informed consent were not required.

**Table 1:** Study characteristics

Study	Study design	Arthroplasty	Follow-up (mean)	Age at surgery (mean)	Septic arthritis group, *n*	Nonseptic arthritis group, *n*	Diagnosis in nonseptic arthritis group	Surgical method in septic arthritis group	NOS
Bettencourt 2021 [9]	Case–control	TKA	9 years	Septic: 62.9 years Nonseptic: 63.9 years	215	215	OA	One-stage TKA: 175 Two-stage TKA: 40	8
Bettencourt 2022 [10]	Case–control	THA	11 years	Septic: 57.8 years Nonseptic: 58.8 years	256	256	OA	One-stage THA: 174 Two-stage THA: 82	8
Dubin 2023 [13]	Retrospective cohort	THA	2 years	Septic: 60 years Nonseptic: 65 years	1052	5000	N/A	N/A	7
Hameed 2023 [16]	Retrospective cohort	TKA	2 years	63–66 years	4251	5000	N/A	N/A	7
Kwon 2025 [17]	Retrospective cohort	TKA	Septic: 45.1 months Nonseptic: 45.8 months	Septic: 73.3 years Nonseptic: 73.2 years	25	75	OA	Two-stage TKA: 25	8
Park 2020 [15]	Retrospective cohort	THA	12.3 years	Septic: 41.2 years Nonseptic: 38.7 years	25	25	DDH	THA with subtrochanteric shortening osteotomy: 25	7
Papanna 2018 [14]	Case–control	THA	Septic: 70 months Nonseptic: 72 months	Septic: 58 years Nonseptic: 57 years	18	18	OA	One-stage THA: 7 Two-stage THA: 11	6

*DDH* developmental dysplasia of the hip, * NOS* Newcastle–Ottawa scale, * OA* osteoarthritis, * THA* total hip arthroplasty * TKA* total knee arthroplasty

### Study selection

This meta-analysis included case–control or cohort studies comparing the risk of postoperative infection, aseptic revision, or reoperation after primary TJA between patients with a history of native knee or hip septic arthritis (septic arthritis group) and those without (nonseptic arthritis group). The septic arthritis group included patients who underwent TJA for arthritic changes caused by sequelae of childhood joint infection and those who underwent one- or two-stage TJA for active or previously treated septic arthritis.

Infection was defined as deep infection or PJI. Aseptic revision was defined as any revision procedure involving component exchange performed for noninfectious causes, including mechanical loosening, instability, wear, and periprosthetic fracture. Studies evaluating revision procedures performed for PJI were excluded from the analysis. Reoperation was defined as any subsequent surgical procedure performed on the index joint after the primary arthroplasty, irrespective of the underlying indication. Both infection-related and aseptic procedures were included in this study.

Studies that did not distinguish wound problems or superficial infection from deep infection or PJI when evaluating infection rates after primary TKA; those that reported the outcomes of TJA only in the septic arthritis group, without comparison to a nonseptic arthritis group (case series); and those that focused on partial replacement arthroplasty were excluded from the analysis.

### Assessment of methodological quality

Two reviewers independently assessed the methodological quality of the selected studies using the Newcastle–Ottawa Scale (NOS), a tool designed to assess the methodological quality of nonrandomized studies in systematic reviews and/or meta-analyses. Further, it comprises three domains: selection of the study groups, comparability of the groups, and ascertainment of either the exposure or outcomes of interest for case–control or cohort studies. The maximum score is 9, and studies with scores of ≥ 7 were considered to be of high methodological quality [4, 11]. Any disagreements between reviewers were resolved via a consensus decision. If a consensus could not be reached, discrepancies were resolved via a discussion with a third investigator.

### Data extraction

Two reviewers independently extracted data from the included studies using a predefined data extraction form. The following information was collected: first author, year of publication, sample size, mean age at the time of surgery, mean follow-up duration, type of TJA (one- or two-stage surgery) in the septic arthritis group, and postoperative outcomes, including infection, aseptic revision, and reoperation.

### Statistical analysis

To evaluate the risk of postoperative complications in the septic arthritis and nonseptic arthritis groups, data on sample size and the number of cases of postoperative infection, aseptic revision, and reoperation in each group were extracted from each study that was included in this meta-analysis. Meta-analyses were performed using a random-effects model, and risk ratios with 95% confidence intervals were calculated. The meta-analysis was conducted for the following outcomes: (1) risk of PJI, (2) risk of aseptic revision, and (3) risk of reoperation.

Statistical heterogeneity among studies was assessed using the *I*^2^ statistic, with *I*^2^ values of 25%, 50%, and 75% representing low, moderate, and high heterogeneity, respectively. Forest plots were used to present the results of individual studies, pooled estimates of effect, and overall summary effects.

Considering the suggested inefficiency of funnel plots for assessing publication bias in the meta-analyses of proportion studies with low event rates, as previously reported, publication bias was not formally assessed [12]. Statistical significance was set at a *p* value of < 0.05. All analyses were performed using Review Manager version 5.3 (Copenhagen, the Nordic Cochrane Centre, The Cochrane Collaboration, 2014).

## Results

### Study selection

**Fig. 1:**
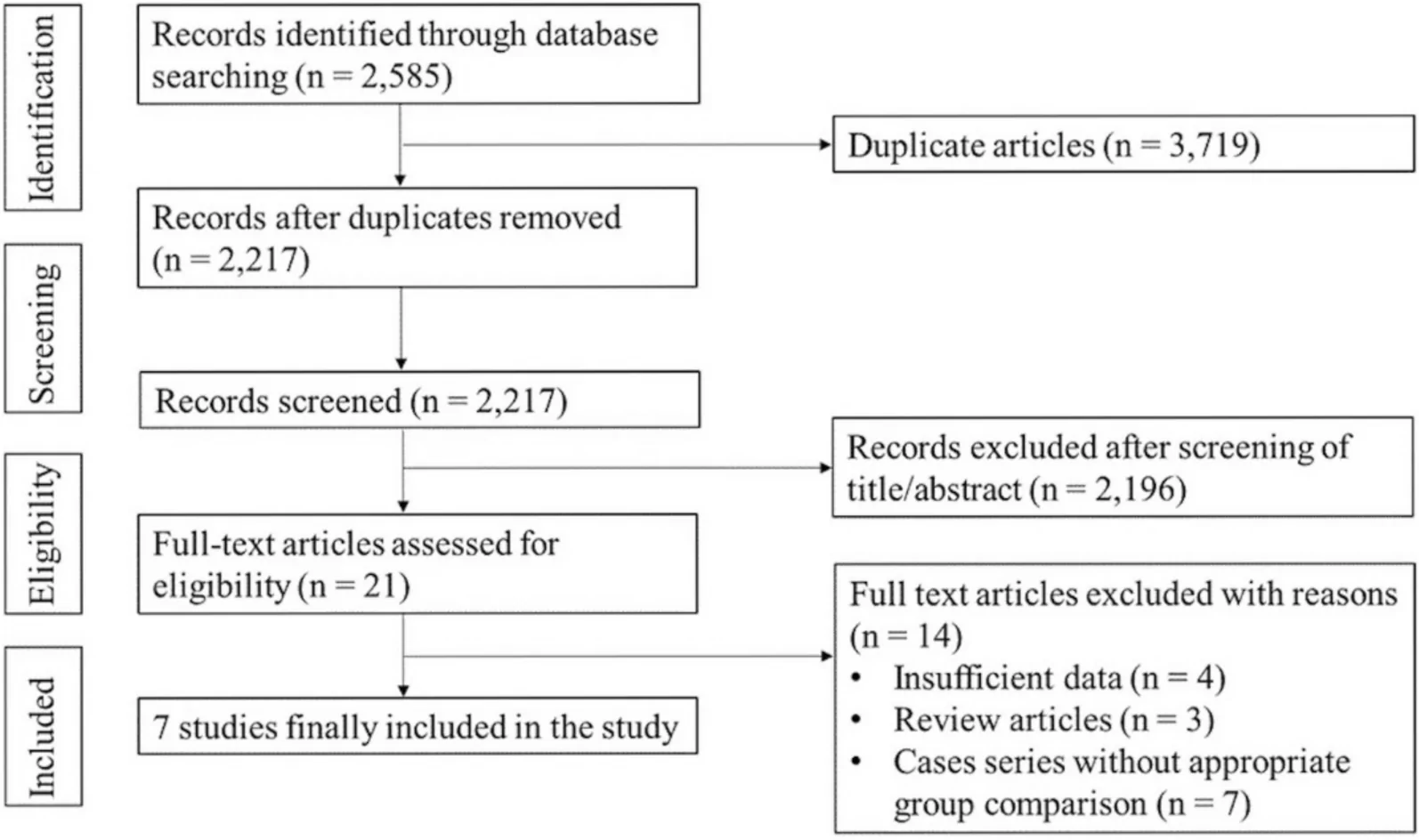
PRISMA flow diagram

Figure 1 shows the study selection process. The literature search identified 2585 records, including 1233 from PubMed (MEDLINE), 1316 from EMBASE, and 36 from the Cochrane Library. No additional relevant studies were identified via manual searching of reference lists. After removing 368 duplicate records, 2217 studies remained for title and abstract screening, of which 2196 were excluded. The remaining 21 studies underwent full-text review. Ultimately, seven studies were included in this systematic review and meta-analysis.

### Characteristics of the studies and risk-of-bias assessment

Across the seven included studies, the septic arthritis group comprised 5842 cases (THA: 1351; TKA: 4491), and the nonseptic arthritis group included 10,589 cases (THA: 5299; TKA: 5290). The mean follow-up duration after TJA in the included studies ranged from 45.1 months to 12.3 years. The mean age of the patients who underwent THA ranged from 38.7 to 58 years. Meanwhile, the mean age of the patients who underwent TKA ranged from 63 to 73.2 years. Five studies reported the causative organisms of native septic arthritis, with *Staphylococcus aureus* being the most commonly identified pathogen.

Four studies evaluated the outcomes of THA between the septic arthritis group and the nonseptic arthritis group [10, 13–15]. One study compared the outcomes of THA between patients with hip dislocation secondary to childhood septic arthritis and those with Crowe type IV developmental dysplasia of the hip [15]. The remaining two studies compared the outcomes of THA between patients with a history of septic hip arthritis and those with hip osteoarthritis [10, 14]. In addition, one study did not report the underlying diagnosis leading to THA [13]. Three studies described the surgical method for THA performed on the septic arthritis group (*n* = 299). In these studies, 206 and 93 patients underwent one- and two-stage THA, respectively [10, 14, 15].

Three studies evaluated the outcomes of TKA between the septic arthritis and nonseptic arthritis groups [9, 16, 17]. Two studies compared patients with a history of septic knee arthritis and those with knee osteoarthritis [9, 17]. Meanwhile, one study did not report the underlying diagnosis leading to TKA in the nonseptic arthritis group [16]. Two studies described the surgical method for TKA performed on the septic arthritis group (*n* = 290). In these studies, 175 and 115 patients underwent one- and two-stage TKA, respectively [9, 17]. Table 1 presents the characteristics of the included studies and the risk-of-bias assessment performed using the Newcastle–Ottawa Scale criteria.

### Comparison of PJI, aseptic revision, and reoperation between the septic arthritis and nonseptic arthritis groups

**Fig. 2:**
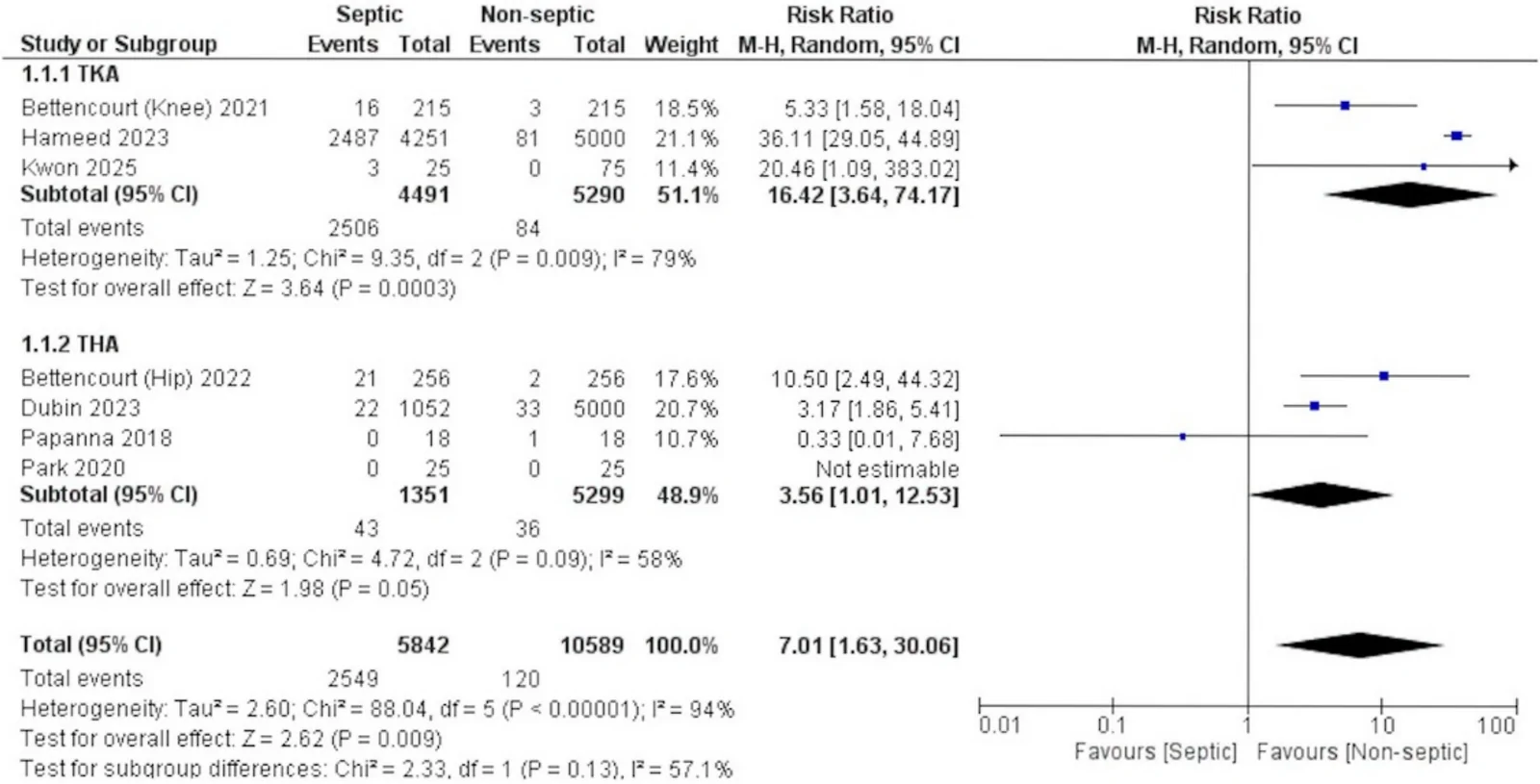
Forest plot comparing the risk of periprosthetic joint infection after total joint arthroplasty in patients with and without prior septic arthritis. M-H = Mantel–Haenszel, and d.f. = degrees of freedom

In total, seven studies were included in the analysis of PJI. Overall, patients with a history of septic arthritis had a significantly higher risk of postoperative PJI than those without (risk ratio [RR] = 7.01, 95% confidence interval [CI]: 1.63–30.06, *p* = 0.009), with substantial heterogeneity among studies (*I*^2^ = 94%) (Fig. 2). In the subgroup analysis, the septic arthritis group was at significantly higher risk of PJI after TKA than the nonseptic arthritis group (RR = 16.4, 95% CI: 3.64–74.17, *p* = 0.0003, *I*^2^ = 79%). In contrast, although patients with a history of septic arthritis undergoing THA exhibited a trend toward an increased risk of PJI, this difference did not reach statistical significance (RR = 3.56, 95% CI: 1.01–12.53, *p* = 0.05, *I*^2^ = 58%).

**Fig. 3:**
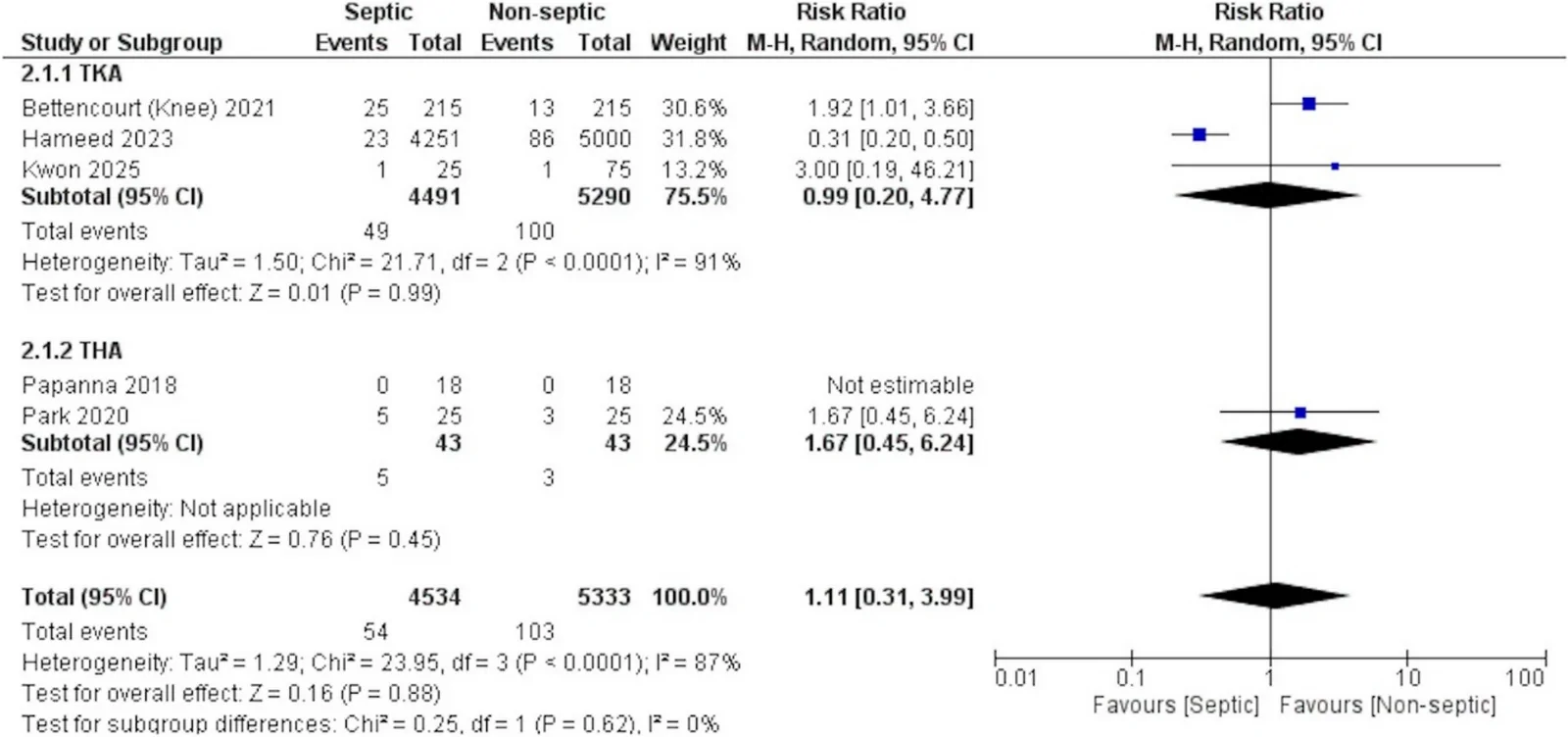
Forest plot comparing the risk of aseptic revision after total joint arthroplasty in patients with and without prior septic arthritis. M-H = Mantel–Haenszel, and d.f. = degrees of freedom

This analysis included five studies reporting aseptic revision. There was no significant difference in the overall risk of aseptic revision between the septic arthritis and nonseptic arthritis groups (RR = 1.11, 95% CI: 0.31–3.99, *p* = 0.88, *I*^2^ = 87%) (Fig. 3). Based on the subgroup analysis, the risk of aseptic revision did not significantly differ between the TKA and THA subgroups.

**Fig. 4:**
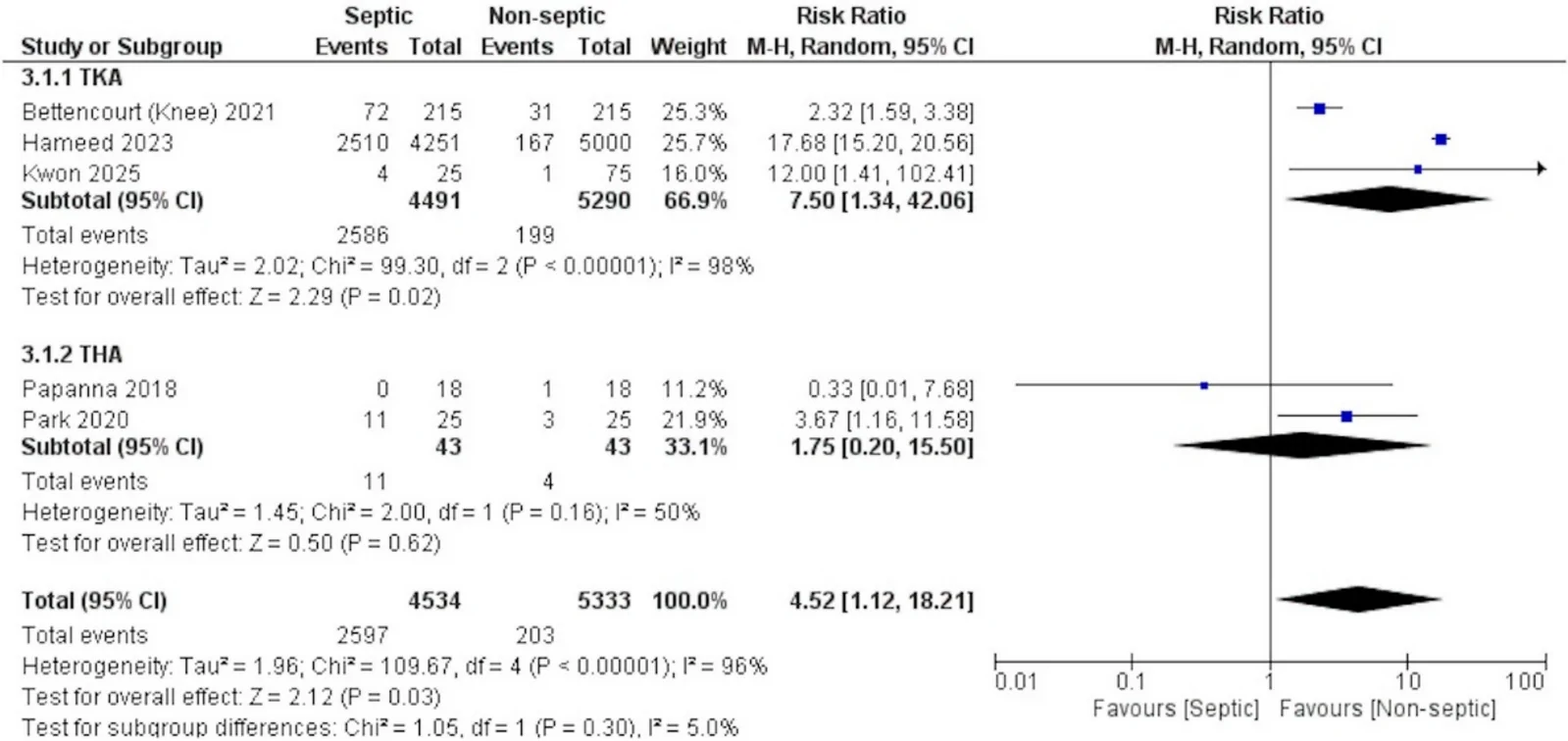
Forest plot comparing the risk of reoperation after total joint arthroplasty in patients with and without prior septic arthritis. M-H = Mantel–Haenszel, and d.f. = degrees of freedom

Five studies were included in the analysis of reoperation. Patients with a history of septic arthritis were at significantly higher risk of reoperation than those without (RR = 4.52, 95% CI: 1.12–18.21, *p* = 0.03), with considerable heterogeneity (*I*^2^ = 96%) (Fig. 4). In a subgroup analysis, a significantly increased risk of reoperation was observed after TKA (RR = 7.50, 95% CI, 1.34–42.06, *p* = 0.02, *I*^2^ = 98%). Meanwhile, there was no statistically significant increase observed after THA (RR = 1.75, 95% CI: 0.20–15.50, *p* = 0.62, *I*^2^ = 50%).

## Discussion

The principal finding of this study is that patients with a history of native hip or knee septic arthritis were at higher risk of postoperative infection and reoperation after TJA compared with those without.

Septic arthritis can lead to the destruction of articular cartilage and subsequent functional impairment if diagnosis or treatment is delayed [1]. Open or arthroscopic debridement is commonly performed for managing septic arthritis. However, as a sequela of septic arthritis, progressive degenerative changes may develop over time. Hence, TJA is ultimately required. In addition, with the increasing proportion of older adults and patients who are immunocompromised, the incidence of septic arthritis occurring in joints with preexisting advanced arthritis has been increasing [2, 3]. In such cases of infected arthritic knees or hips, two-stage TJA using an antibiotic-impregnated articulating or static cement spacer has also been performed [5–7]. Regardless of the timing of native joint infection, patients with a history of septic arthritis who underwent TJA may exhibit outcomes that differ from those of patients without such a history, particularly with respect to the risk of postoperative complications, such as infection.

The most consistent finding of this meta-analysis was that patients with a history of septic arthritis were at increased risk of PJI after TJA. This association was observed across most included studies and was evident after TKA, with a substantially higher relative risk reported. Bettencourt et al. [9] revealed a significantly increased risk of infection after TKA performed on knees with a previous history of septic arthritis. Further, their study showed that this elevated risk was more likely attributed to alterations in the local joint environment rather than to systemic factors. The pathophysiology of implant-associated infection has revealed that bacteria can persist in a dormant state or within biofilms even if infection appears clinically resolved, and that prosthesis implantation may promote the reactivation of these residual microorganisms [18, 19]. In addition, several studies have reported that the local joint environment may remain altered for prolonged periods after septic arthritis. Shirtliff and Mader [20] found persistent inflammatory activity and altered immune responses within synovial and intraarticular tissues even after apparent resolution of infection. Meanwhile, Masters et al.[21] showed that long-term changes in host–pathogen interactions after osteoarticular infection may adversely affect the joint microenvironment. Taken together, multiple factors—including residual bacteria and dormant biofilms within periarticular tissues, sustained alterations in local immune responses. Further, structural damage to the soft tissues and bone—may contribute to an increased risk of PJI after prosthetic implantation. Accordingly, joints with a previous history of septic arthritis should be considered as biologically distinct from joints undergoing TJA for primary degenerative conditions.

A subgroup analysis showed a more pronounced increase in the risk of PJI after TKA compared with THA. These findings indicate that the observed differences in the risk of PJI may be related to joint-specific factors rather than a direct comparative effect between procedures. Joint-specific factors may include differences in local anatomy, soft-tissue coverage, vascularity, and the extent of surgical exposure, all of which can affect susceptibility to infection [19, 22, 23]. Nevertheless, the limited number of studies evaluating THA should be considered when interpreting these findings, as the statistical power of the THA subgroup analysis might have been insufficient.

In this meta-analysis, a history of septic arthritis was not significantly associated with an increased risk of aseptic revision. Although septic arthritis increases susceptibility to postoperative infection, it may not adversely affect mechanical outcomes once infection is adequately controlled. However, the conditions that typically require aseptic revision—such as loosening, wear, instability, and periprosthetic fracture—are relatively rare events, and their accurate assessment requires long-term follow-up. This might have limited the ability of individual studies to detect minimal differences in mechanical failure rates between groups.

In contrast, patients with a history of septic arthritis are at significantly higher risk of reoperation. In the included studies, reoperation encompassed a broad range of additional surgical procedures, including infection-related surgeries, irrigation, debridement, and closed reduction for dislocation under anesthesia. The aseptic revision rates did not significantly differ between patients with a history of septic arthritis and those without. However, patients with a previous history of septic arthritis more frequently underwent additional surgical procedures. Therefore, the higher reoperation rate is likely explained, at least in part, by the substantially increased incidence of PJI and the resulting need for infection-related surgical procedures in patients with a history of septic arthritis.

This meta-analysis has several limitations. First, most of the included studies were retrospective in nature. Therefore, the influence of residual confounding factors could not be completely excluded. Second, the definitions of reoperation and aseptic revision were not completely uniform across the included studies. Database-based studies provided limited information regarding the specific types and extent of surgical procedures performed. Third, not all studies reported aseptic revision and reoperation as separate outcomes, which reduced the number of studies available for inclusion in each individual analysis. Fourth, substantial heterogeneity was observed across the pooled analyses. This heterogeneity likely reflects the considerable clinical and methodological diversity among the included studies, including differences in patient populations, comparator diagnoses, timing of arthroplasty after septic arthritis, surgical strategies (one-stage versus two-stage procedures), outcome definitions, and duration of follow-up. Because only seven comparative studies were eligible for inclusion, further analyses such as meta-regression were not considered appropriate. Therefore, the pooled estimates should be interpreted with appropriate caution. Finally, the number of studies included in the THA subgroup analysis was limited. Therefore, the study results should be interpreted with caution.

Despite these limitations, comparative studies evaluating outcomes after TJA in patients with a history of native hip or knee septic arthritis remain limited. In this context, to our knowledge, the present study is the first systematic review and meta-analysis to quantitatively synthesize the available comparative evidence evaluating the outcomes of TJA in patients with a history of native septic arthritis. The findings provide a quantitative summary of the currently available comparative evidence and may help inform future research and clinical interpretation of the available literature.

## Conclusions

Patients with a history of native hip or knee septic arthritis are at significantly higher risk of postoperative infection and reoperation after total joint arthroplasty compared with those without a history of joint infection. Surgeons should be aware of the increased risk of complications in this high-risk population. Nevertheless, further studies should be performed to determine the optimal timing of arthroplasty after septic arthritis and to identify strategies that can reduce postoperative complications.

## Supplementary Information


Supplementary Material 1

## Data Availability

The datasets generated and/or analyzed during the current study are available from the corresponding author on reasonable request.

## Abbreviations

CI: confidence interval
PJI: periprosthetic joint infection
RR: risk ratio
THA: total hip arthroplasty
TJA: total joint arthroplasty
TKA: total knee arthroplasty
