# Supplementary material for: Outcomes of total joint arthroplasty in patients with a history of native hip or knee septic arthritis: a systematic review and meta-analysis
Source: Knee Surg Relat Res. 2026 Aug 31;38:45. doi: 10.1186/s43019-026-00348-y (PMC13528027; doi:10.1186/s43019-026-00348-y)
Supplement: Supplementary file 1 [file 43019_2026_348_MOESM1_ESM.docx]

Supplementary file

Appendix Table 1. Search protocol

| Database | No. | Search query |
| --- | --- | --- |
| PubMed | #1 | "Arthroplasty, Replacement, Knee"[Mesh] |
|  | #2 | "Arthroplasty, Replacement, Knee"[TW] OR "Arthroplasties, Replacement, Knee"[TW] OR "Arthroplasty, Knee Replacement"[TW] OR "Knee Replacement Arthroplasties"[TW] OR "Knee Replacement Arthroplasty"[TW] OR "Replacement Arthroplasties, Knee"[TW] OR "Knee Arthroplasty, Total"[TW] OR "Arthroplasty, Total Knee"[TW] OR "Total Knee Arthroplasty"[TW] OR "Replacement, Total Knee"[TW] OR "Total Knee Replacement"[TW] OR "Knee Replacement, Total"[TW] OR "Knee Arthroplasty"[TW] OR "Arthroplasty, Knee"[TW] OR "Arthroplasties, Knee Replacement"[TW] OR "Replacement Arthroplasty, Knee"[TW] OR "total knee joint replacement"[TW] OR "total knee replacement arthroplasty"[TW] OR "TKA"[TW] OR "TKAs"[TW] OR "total knee arthroplasties"[TW] OR "TKR"[TW] OR "knee replacement"[TW] OR "knee arthroplasties"[TW] |
|  | #3 | "Arthroplasty, Replacement, Hip"[Mesh] |
|  | #4 | "Arthroplasty, Replacement, Hip"[TW] OR "Arthroplasties, Replacement, Hip"[TW] OR "Arthroplasty, Hip Replacement"[TW] OR "Hip Replacement Arthroplasties"[TW] OR "Hip Prosthesis Implantation"[TW] OR "Hip Prosthesis Implantations"[TW] OR "Implantation, Hip Prosthesis"[TW] OR "Prosthesis Implantation, Hip"[TW] OR "Replacement Arthroplasties, Hip"[TW] OR "Replacement Arthroplasty, Hip"[TW] OR "Arthroplasties, Hip Replacement"[TW] OR "Hip Replacement Arthroplasty"[TW] OR "Hip Replacement, Total"[TW] OR "Replacement, Total Hip"[TW] OR "Total Hip Replacements"[TW] OR "Total Hip Replacement"[TW] OR "Total Hip Arthroplasty"[TW] OR "Arthroplasty, Total Hip"[TW] OR "Hip Arthroplasty, Total"[TW] OR "Total Hip Arthroplasties"[TW] OR "hip total arthroplasty"[TW] OR "hip total joint replacement"[TW] OR "hip total replacement"[TW] OR "hip total replacement arthroplasty"[TW] OR "total replacement hip arthroplasty"[TW] OR "THA"[TW] OR "THAs"[TW] OR "THR"[TW] OR "hip replacement"[TW] OR "hip arthroplasty"[TW] OR "hip arthroplasties"[TW] |
|  | #5 | #1 OR #2 OR #3 OR #4 |
|  | #6 | "Arthritis, Infectious"[Mesh] |
|  | #7 | "Arthritis, Infectious"[TW] OR "Infectious Arthritis"[TW] OR "Arthritis, Viral"[TW] OR "Viral Arthritis"[TW] OR "Arthritis, Bacterial"[TW] OR "Bacterial Arthritides"[TW] OR "Arthritis, Septic"[TW] OR "Septic Arthritis"[TW] OR "Arthritides, Bacterial"[TW] OR "Bacterial Arthritis"[TW] OR "Arthritis, Suppurative"[TW] OR "Suppurative Arthritis"[TW] OR "arthritis, purulent"[TW] OR "purulent arthritis"[TW] OR "pyarthrosis"[TW] OR "pyoarthritis"[TW] OR "pyogenic arthritis"[TW] OR "infective arthritis"[TW] |
|  | #8 | #6 OR #7 |
|  | #9 | "Infections"[Mesh] |
|  | #10 | "Infections"[TW] OR "Infection and Infestation"[TW] OR "Infestation and Infection"[TW] OR "Infections and Infestations"[TW] OR "Infestations and Infections"[TW] OR "Infection"[TW] OR "infectious disease"[TW] OR "infectivity"[TW] OR "postopertive infection"[TW] OR "Healthcare-associated infection"[TW] OR "HAI"[TW] |
|  | #11 | "Prosthesis-Related Infections"[Mesh] |
|  | #12 | "Prosthesis-Related Infections"[TW] OR "Prosthesis Related Infections"[TW] OR "Prosthesis Related Infection"[TW] OR "Infection, Prosthesis Related"[TW] OR "Related Infection, Prosthesis"[TW] OR "Related Infections, Prosthesis"[TW] OR "Prosthesis-Related Infection"[TW] OR "Infections, Prosthesis-Related"[TW] OR "prosthesis infection"[TW] OR "prosthesis-associated infection"[TW] OR "prosthetic device infection"[TW] OR "prosthetic infection"[TW] OR "periprosthetic joint infection"[TW] OR "PJI"[TW] |
|  | #13 | #9 OR #10 OR #11 OR #12 |
|  | #14 | #5 AND #8 AND #13 |
|  | #15 | (#1 OR #2) AND #8 AND #13 |
|  | #16 | (#3 OR #4) AND #8 AND #13 |

| Database | No. | Search query |
| --- | --- | --- |
| Embase via elsevier | #1 | "total knee arthroplasty"/mj |
|  | #2 | "Arthroplasty, Replacement, Knee":ti,ab,kw,de OR "Arthroplasties, Replacement, Knee":ti,ab,kw,de OR "Arthroplasty, Knee Replacement":ti,ab,kw,de OR "Knee Replacement Arthroplasties":ti,ab,kw,de OR "Knee Replacement Arthroplasty":ti,ab,kw,de OR "Replacement Arthroplasties, Knee":ti,ab,kw,de OR "Knee Arthroplasty, Total":ti,ab,kw,de OR "Arthroplasty, Total Knee":ti,ab,kw,de OR "Total Knee Arthroplasty":ti,ab,kw,de OR "Replacement, Total Knee":ti,ab,kw,de OR "Total Knee Replacement":ti,ab,kw,de OR "Knee Replacement, Total":ti,ab,kw,de OR "Knee Arthroplasty":ti,ab,kw,de OR "Arthroplasty, Knee":ti,ab,kw,de OR "Arthroplasties, Knee Replacement":ti,ab,kw,de OR "Replacement Arthroplasty, Knee":ti,ab,kw,de OR "total knee joint replacement":ti,ab,kw,de OR "total knee replacement arthroplasty":ti,ab,kw,de OR "TKA":ti,ab,kw,de OR "TKAs":ti,ab,kw,de OR "total knee arthroplasties":ti,ab,kw,de OR "TKR":ti,ab,kw,de OR "knee replacement":ti,ab,kw,de OR "knee arthroplasties":ti,ab,kw,de |
|  | #3 | "total hip replacement"/mj |
|  | #4 | "Arthroplasty, Replacement, Hip":ti,ab,kw,de OR "Arthroplasties, Replacement, Hip":ti,ab,kw,de OR "Arthroplasty, Hip Replacement":ti,ab,kw,de OR "Hip Replacement Arthroplasties":ti,ab,kw,de OR "Hip Prosthesis Implantation":ti,ab,kw,de OR "Hip Prosthesis Implantations":ti,ab,kw,de OR "Implantation, Hip Prosthesis":ti,ab,kw,de OR "Prosthesis Implantation, Hip":ti,ab,kw,de OR "Replacement Arthroplasties, Hip":ti,ab,kw,de OR "Replacement Arthroplasty, Hip":ti,ab,kw,de OR "Arthroplasties, Hip Replacement":ti,ab,kw,de OR "Hip Replacement Arthroplasty":ti,ab,kw,de OR "Hip Replacement, Total":ti,ab,kw,de OR "Replacement, Total Hip":ti,ab,kw,de OR "Total Hip Replacements":ti,ab,kw,de OR "Total Hip Replacement":ti,ab,kw,de OR "Total Hip Arthroplasty":ti,ab,kw,de OR "Arthroplasty, Total Hip":ti,ab,kw,de OR "Hip Arthroplasty, Total":ti,ab,kw,de OR "Total Hip Arthroplasties":ti,ab,kw,de OR "hip total arthroplasty":ti,ab,kw,de OR "hip total joint replacement":ti,ab,kw,de OR "hip total replacement":ti,ab,kw,de OR "hip total replacement arthroplasty":ti,ab,kw,de OR "total replacement hip arthroplasty":ti,ab,kw,de OR "THA":ti,ab,kw,de OR "THAs":ti,ab,kw,de OR "THR":ti,ab,kw,de OR "hip replacement":ti,ab,kw,de OR "hip arthroplasty":ti,ab,kw,de OR "hip arthroplasties":ti,ab,kw,de |
|  | #5 | #1 OR #2 OR #3 OR #4 |
|  | #6 | "infectious arthritis"/mj OR "bacterial arthritis"/mj |
|  | #7 | "Arthritis, Infectious":ti,ab,kw,de OR "Infectious Arthritis":ti,ab,kw,de OR "Arthritis, Viral":ti,ab,kw,de OR "Viral Arthritis":ti,ab,kw,de OR "Arthritis, Bacterial":ti,ab,kw,de OR "Bacterial Arthritides":ti,ab,kw,de OR "Arthritis, Septic":ti,ab,kw,de OR "Septic Arthritis":ti,ab,kw,de OR "Arthritides, Bacterial":ti,ab,kw,de OR "Bacterial Arthritis":ti,ab,kw,de OR "Arthritis, Suppurative":ti,ab,kw,de OR "Suppurative Arthritis":ti,ab,kw,de OR "arthritis, purulent":ti,ab,kw,de OR "purulent arthritis":ti,ab,kw,de OR "pyarthrosis":ti,ab,kw,de OR "pyoarthritis":ti,ab,kw,de OR "pyogenic arthritis":ti,ab,kw,de OR "infective arthritis":ti,ab,kw,de |
|  | #8 | #6 OR #7 |
|  | #9 | "infection"/mj |
|  | #10 | "Infections":ti,ab,kw,de OR "Infection and Infestation":ti,ab,kw,de OR "Infestation and Infection":ti,ab,kw,de OR "Infections and Infestations":ti,ab,kw,de OR "Infestations and Infections":ti,ab,kw,de OR "Infection":ti,ab,kw,de OR "infectious disease":ti,ab,kw,de OR "infectivity":ti,ab,kw,de OR "postopertive infection":ti,ab,kw,de OR "Healthcare-associated infection":ti,ab,kw,de OR "HAI":ti,ab,kw,de |
|  | #11 | "prosthesis infection"/mj OR "periprosthetic joint infection"/mj |
|  | #12 | "Prosthesis-Related Infections":ti,ab,kw,de OR "Prosthesis Related Infections":ti,ab,kw,de OR "Prosthesis Related Infection":ti,ab,kw,de OR "Infection, Prosthesis Related":ti,ab,kw,de OR "Related Infection, Prosthesis":ti,ab,kw,de OR "Related Infections, Prosthesis":ti,ab,kw,de OR "Prosthesis-Related Infection":ti,ab,kw,de OR "Infections, Prosthesis-Related":ti,ab,kw,de OR "prosthesis infection":ti,ab,kw,de OR "prosthesis-associated infection":ti,ab,kw,de OR "prosthetic device infection":ti,ab,kw,de OR "prosthetic infection":ti,ab,kw,de OR "periprosthetic joint infection":ti,ab,kw,de OR "PJI":ti,ab,kw,de |
|  | #13 | #9 OR #10 OR #11 OR #12 |
|  | #14 | #5 AND #8 AND #13 |
|  | #15 | (#1 OR #2) AND #8 AND #13 |
|  | #16 | (#3 OR #4) AND #8 AND #13 |

| Database | No. | Search query |
| --- | --- | --- |
| Cochrane Library | #1 | [mh "Arthroplasty, Replacement, Knee"] |
|  | #2 | "Arthroplasty, Replacement, Knee":ti,ab,kw OR "Arthroplasties, Replacement, Knee":ti,ab,kw OR "Arthroplasty, Knee Replacement":ti,ab,kw OR "Knee Replacement Arthroplasties":ti,ab,kw OR "Knee Replacement Arthroplasty":ti,ab,kw OR "Replacement Arthroplasties, Knee":ti,ab,kw OR "Knee Arthroplasty, Total":ti,ab,kw OR "Arthroplasty, Total Knee":ti,ab,kw OR "Total Knee Arthroplasty":ti,ab,kw OR "Replacement, Total Knee":ti,ab,kw OR "Total Knee Replacement":ti,ab,kw OR "Knee Replacement, Total":ti,ab,kw OR "Knee Arthroplasty":ti,ab,kw OR "Arthroplasty, Knee":ti,ab,kw OR "Arthroplasties, Knee Replacement":ti,ab,kw OR "Replacement Arthroplasty, Knee":ti,ab,kw OR "total knee joint replacement":ti,ab,kw OR "total knee replacement arthroplasty":ti,ab,kw OR "TKA":ti,ab,kw OR "TKAs":ti,ab,kw OR "total knee arthroplasties":ti,ab,kw OR "TKR":ti,ab,kw OR "knee replacement":ti,ab,kw OR "knee arthroplasties":ti,ab,kw |
|  | #3 | [mh "Arthroplasty, Replacement, Hip"] |
|  | #4 | "Arthroplasty, Replacement, Hip":ti,ab,kw OR "Arthroplasties, Replacement, Hip":ti,ab,kw OR "Arthroplasty, Hip Replacement":ti,ab,kw OR "Hip Replacement Arthroplasties":ti,ab,kw OR "Hip Prosthesis Implantation":ti,ab,kw OR "Hip Prosthesis Implantations":ti,ab,kw OR "Implantation, Hip Prosthesis":ti,ab,kw OR "Prosthesis Implantation, Hip":ti,ab,kw OR "Replacement Arthroplasties, Hip":ti,ab,kw OR "Replacement Arthroplasty, Hip":ti,ab,kw OR "Arthroplasties, Hip Replacement":ti,ab,kw OR "Hip Replacement Arthroplasty":ti,ab,kw OR "Hip Replacement, Total":ti,ab,kw OR "Replacement, Total Hip":ti,ab,kw OR "Total Hip Replacements":ti,ab,kw OR "Total Hip Replacement":ti,ab,kw OR "Total Hip Arthroplasty":ti,ab,kw OR "Arthroplasty, Total Hip":ti,ab,kw OR "Hip Arthroplasty, Total":ti,ab,kw OR "Total Hip Arthroplasties":ti,ab,kw OR "hip total arthroplasty":ti,ab,kw OR "hip total joint replacement":ti,ab,kw OR "hip total replacement":ti,ab,kw OR "hip total replacement arthroplasty":ti,ab,kw OR "total replacement hip arthroplasty":ti,ab,kw OR "THA":ti,ab,kw OR "THAs":ti,ab,kw OR "THR":ti,ab,kw OR "hip replacement":ti,ab,kw OR "hip arthroplasty":ti,ab,kw OR "hip arthroplasties":ti,ab,kw |
|  | #5 | #1 OR #2 OR #3 OR #4 |
|  | #6 | [mh "Arthritis, Infectious"] |
|  | #7 | "Arthritis, Infectious":ti,ab,kw OR "Infectious Arthritis":ti,ab,kw OR "Arthritis, Viral":ti,ab,kw OR "Viral Arthritis":ti,ab,kw OR "Arthritis, Bacterial":ti,ab,kw OR "Bacterial Arthritides":ti,ab,kw OR "Arthritis, Septic":ti,ab,kw OR "Septic Arthritis":ti,ab,kw OR "Arthritides, Bacterial":ti,ab,kw OR "Bacterial Arthritis":ti,ab,kw OR "Arthritis, Suppurative":ti,ab,kw OR "Suppurative Arthritis":ti,ab,kw OR "arthritis, purulent":ti,ab,kw OR "purulent arthritis":ti,ab,kw OR "pyarthrosis":ti,ab,kw OR "pyoarthritis":ti,ab,kw OR "pyogenic arthritis":ti,ab,kw OR "infective arthritis":ti,ab,kw |
|  | #8 | #6 OR #7 |
|  | #9 | [mh "Infections"] |
|  | #10 | "Infections":ti,ab,kw OR "Infection and Infestation":ti,ab,kw OR "Infestation and Infection":ti,ab,kw OR "Infections and Infestations":ti,ab,kw OR "Infestations and Infections":ti,ab,kw OR "Infection":ti,ab,kw OR "infectious disease":ti,ab,kw OR "infectivity":ti,ab,kw OR "postopertive infection":ti,ab,kw OR "Healthcare-associated infection":ti,ab,kw OR "HAI":ti,ab,kw |
|  | #11 | [mh "Prosthesis-Related Infections"] |
|  | #12 | "Prosthesis-Related Infections":ti,ab,kw OR "Prosthesis Related Infections":ti,ab,kw OR "Prosthesis Related Infection":ti,ab,kw OR "Infection, Prosthesis Related":ti,ab,kw OR "Related Infection, Prosthesis":ti,ab,kw OR "Related Infections, Prosthesis":ti,ab,kw OR "Prosthesis-Related Infection":ti,ab,kw OR "Infections, Prosthesis-Related":ti,ab,kw OR "prosthesis infection":ti,ab,kw OR "prosthesis-associated infection":ti,ab,kw OR "prosthetic device infection":ti,ab,kw OR "prosthetic infection":ti,ab,kw OR "periprosthetic joint infection":ti,ab,kw OR "PJI":ti,ab,kw |
|  | #13 | #9 OR #10 OR #11 OR #12 |
|  | #14 | #5 AND #8 AND #13 |
|  | #15 | (#1 OR #2) AND #8 AND #13 |
|  | #16 | (#3 OR #4) AND #8 AND #13 |
